# Antitumor activity of gilteritinib, an inhibitor of AXL, in human solid tumors

**DOI:** 10.1038/s41420-025-02417-9

**Published:** 2025-03-29

**Authors:** Zuxiong Zhang, Ruxia Hu, Jie Liu, Xiaohan Yang, Youban Xiao, Xi Xu, Xinxin Liu, Wen Zeng, Shuyong Zhang, Liefeng Wang

**Affiliations:** 1 Department of Thoracic Surgery, The First Affiliated Hospital, Gannan Medical University, Ganzhou, Jiangxi China; 2 Key Laboratory of Prevention and Treatment of Cardiovascular and Cerebrovascular Diseases (Ministry of Education), Gannan Medical University, Ganzhou, China; 3 School of Basic Medicine, Gannan Medical University, Ganzhou, China; 4 School of Rehabilitation Medicine, Gannan Medical University, Ganzhou, China; 5 Department of Respiratory and Critical Care Medicine, The First Affiliated Hospital, Gannan Medical University, Ganzhou, Jiangxi China; 6 Department of Surgical Oncology, Ganzhou Cancer Hospital, Gannan Medical University, Ganzhou, China

**Keywords:** Chemotherapy, Cancer prevention

## Abstract

AXL, a receptor tyrosine kinase, has recently emerged as a potential therapeutic target against various types of cancer. Gilteritinib, a FDA-approved small-molecule inhibitor, is used for the treatment of patients with FLT3-mutated acute myeloid leukemia. However, the antitumor activity of gilteritinib in solid tumors remains poorly elucidated. In this study, we explored the antitumor activity of gilteritinib in AXL-expressing esophageal cancer (EC), ovarian cancer (OC), and gastric cancer (GC), along with the underlying molecular mechanisms. Our data demonstrated that gilteritinib significantly inhibited cell proliferation and spheroid formation by triggering apoptosis and cell cycle arrest in AXL-positive EC, OC, and GC cells. Moreover, we found that gilteritinib treatment repressed EC, OC, and GC cell migration and invasion. Mechanistically, RNA-seq analysis revealed that gilteritinib significantly downregulated multiple cancer-related pathways, including those related to apoptosis, the cell cycle, the mTOR pathway, the AMPK pathway, the p53 pathway, the FOXO pathway, the Hippo pathway, and the Wnt pathway. Gilteritinib inhibited a unique set of E2F- and MYC target-associated genes in EC, OC, and GC cells. Intriguingly, interrogation of the EC, OC, and GC cohort demonstrated that these genes were overexpressed and associated with poor prognosis. Gilteritinib also displayed strong antitumor effects on AXL-positive PDX-derived explants (PDXEs) and PDX-derived organoids (PDXOs) ex vivo and PDXs in vivo. Collectively, these findings reveal that gilteritinib represents a potent therapeutic agent for the treatment of AXL-positive solid tumors.

**Figure:**
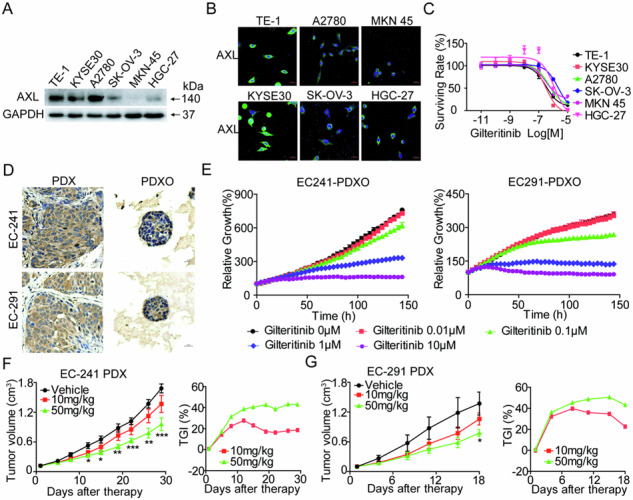
Zhang et al. demonstrate superior therapeutic efficacy of Gilteritinib, a FDA-approved small-molecule inhibitor, in the AXL-expressing esophageal cancer, ovarian cancer and gastric cancer cell lines, PDXOs and PDXs models. This work highlights Gilteritinib as a novel and potent therapeutic approach for the treatment of AXL-positive solid tumors.

Zhang et al. demonstrate superior therapeutic efficacy of Gilteritinib, a FDA-approved small-molecule inhibitor, in the AXL-expressing esophageal cancer, ovarian cancer and gastric cancer cell lines, PDXOs and PDXs models. This work highlights Gilteritinib as a novel and potent therapeutic approach for the treatment of AXL-positive solid tumors.

## Introduction

Cancer is a major public health problem worldwide [1, 2]. It often remains asymptomatic in its early stages and is frequently diagnosed at advanced or metastatic stages, making it difficult to identify the most effective treatment opportunity. Although cancer survival has improved with diagnostic and therapeutic advances, the prognosis of some patients with cancer remains poor. Gastric cancer (GC) is the fifth most common malignant tumor and the fourth leading cause of cancer-related deaths worldwide. The 5-year survival rate of advanced GC is only 17.8% [3]. Esophageal cancer (EC) is the sixth leading cause of cancer-related death worldwide, and the 5-year survival rate for advanced EC is only 20% [4]. Ovarian cancer (OC) is one of the most common malignant tumors of the female reproductive system. The 5-year survival rate for advanced OC is only 49.1% [5, 6]. Therefore, there is an unmet clinical need for new therapeutic strategies for solid tumors.

AXL, a receptor tyrosine kinase and an important member of the TAM family (Tyro3, Axl, Mer), is abnormally overexpressed in various malignant tumors, including EC, GC, OC, and colorectal cancer (CRC) [7]. Notably, AXL was first identified as a transforming gene in chronic myeloid leukemia (CML) [8, 9]. Overexpression or activation of AXL promotes tumor growth, differentiation, proliferation, and metastasis by activating the PI3K/AKT and/or MAPK/ERK pathways [10–12]. Increasing evidence has shown that the overexpression or activation of AXL is associated with metastasis, drug resistance, and poorer prognosis in many cancers [13–16]. Therefore, AXL inhibition has emerged as a promising strategy for developing targeted anticancer agents against various cancers.

Gilteritinib, a pyrazine carboxamide derivative, is an orally available FDA-approved FMS-like tyrosine kinase 3 (FLT3) inhibitor that is used for treating patients with FLT3-mutated acute myeloid leukemia (AML) [17–19]. In addition to FLT3, gilteritinib inhibits AXL, which is frequently overexpressed in AML [20]. Interestingly, AXL facilitates FLT3 activation in AML, which may be potentially involved in drug resistance to other FLT3 inhibitors [21, 22]. Thus, gilteritinib is a small-molecule inhibitor that targets both FLT3 and AXL. Gilteritinib has a good clinical effect and is therefore already approved for relapsed/refractory FLT3-mutated AML [18]. Currently, a phase I study of gilteritinib along with induction and consolidation chemotherapy in patients with newly diagnosed AML is ongoing (NCT02236013) [23]. Furthermore, two phase III studies comparing gilteritinib and midostaurin in combination with standard chemotherapy are under investigation (NCT03836209 and NCT04027309) [24], as well as several clinical trials of gilteritinib combined with hypomethylating agents and/or venetoclax for the treatment of FLT3-mutated AML (NCT02752035, NCT03404193, and NCT05010122) [24]. To date, most clinical studies on gilteritinib have focused on patients with FLT3-mutated AML. However, the antitumor activity of gilteritinib in solid tumors remains poorly studied.

In this study, we investigated the therapeutic efficacy and relevant molecular pathways of gilteritinib in EC, OC, and GC. Our data demonstrated that gilteritinib enhanced the antitumor activity in vitro and ex vivo. Collectively, these findings revealed gilteritinib as a potent therapeutic agent for treating AXL-positive cancers.

## Results

### Gilteritinib significantly inhibits AXL-positive solid tumor cell viability

**Fig. 1:**
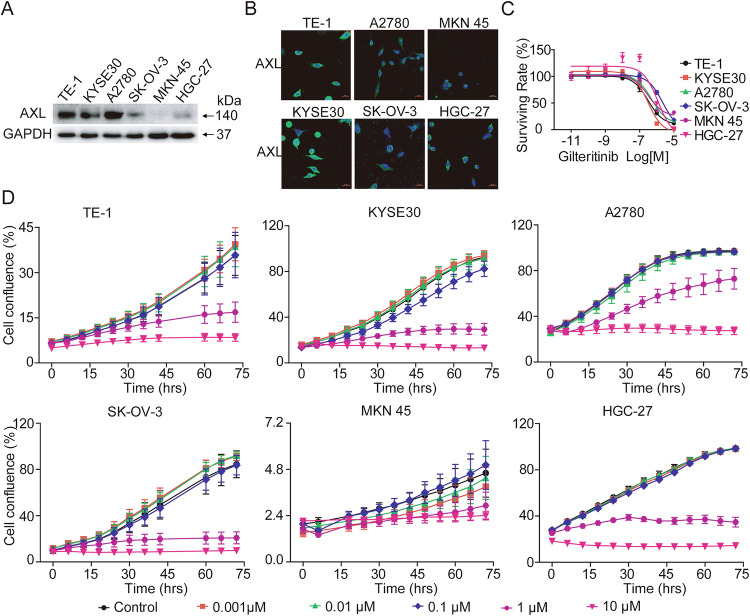
Gilteritinib inhibits AXL-positive solid tumor cell viability. AXL protein expression in KYSE30, TE-1, A2780, SK-OV-3, HGC-27, and MKN45 cells was detected by western blot (**A**) and immunofluorescence staining (**B**). **C** Cells were treated with different concentrations of gilteritinib for 72 h, and the cell viability was measured by Cell Titer-Glo® Luminescent Cell Viability Assay. **D** Cells were treated with gilteritinib at the indicated concentrations. The cell confluency (%) was calculated using Incucyte S3 Zoom software based on phase-contrast images from 0 h to 72 h. Fluorescence images were obtained and analyzed with a laser confocal fluorescence microscope. Scale bars: 20 µm.

To determine the antiproliferative activity of gilteritinib in AXL-positive solid tumors, we observed the expression of AXL in the human EC cell lines KYSE30 and TE-1, OC cell lines A2780 and SK-OV-3, and GC cell lines HGC-27 and MKN45. Western blot and immunofluorescence assays demonstrated that all tested cancer cells expressed AXL, with high levels observed in TE-1, KYSE30, and A2780 cells and relatively low levels in MKN45 cells (Fig. 1A, B). Next, we sought to investigate the effects of gilteritinib on EC, OC, and GC cell proliferation and viability in vitro. The results of the Cell TiterGlo Luminescent Cell Viability assay showed that gilteritinib markedly inhibited the growth of tested human solid tumor cells in a dose-dependent manner. The IC50 values of gilteritinib in KYSE30, TE-1, A2780, SK-OV-3, HGC-27, and MKN45 cells were 1.49 ± 0.84 μM, 0.74 ± 0.30 μM, 0.51 ± 0.01 μM, 2.37 ± 0.09 μM, 0.33 ± 0.02 μM, and 0.34 ± 0.01 μM, respectively (Fig. 1C). In addition, dose- and time-dependent effects of gilteritinib in these cells were measured by cell confluence using the IncuCyte Live Cell Imaging system, and similar dose responses were observed in KYSE30, TE-1, A2780, SK-OV-3, HGC-27, and MKN45 cells (Fig. 1D). These two assays showed that gilteritinib dose- and time-dependently inhibited cell growth, suggesting that gilteritinib had strong antitumor potential in AXL-positive tumors.

### Gilteritinib significantly inhibits cell proliferation and formation in vitro

**Fig. 2:**
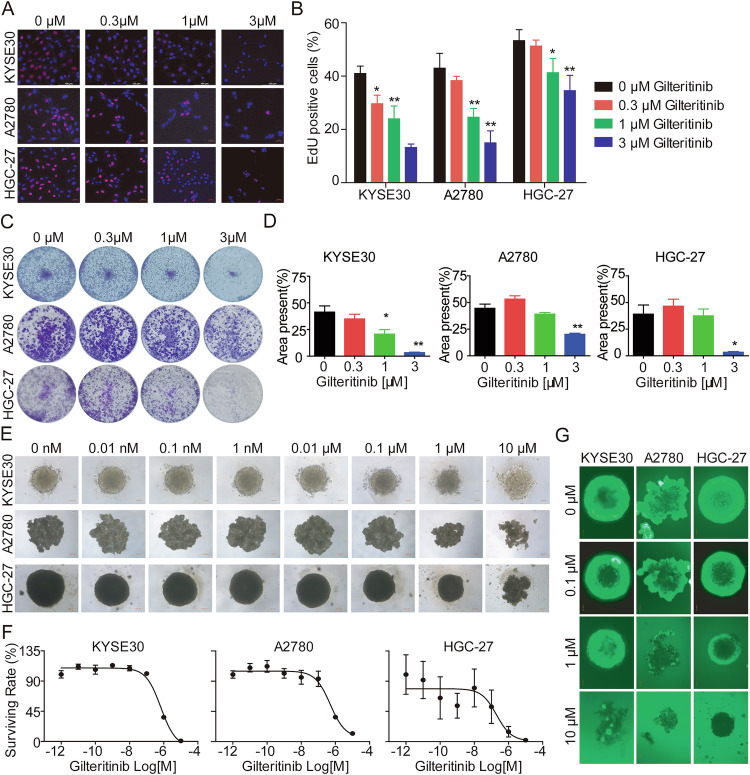
Gilteritinib treatment inhibits cell proliferation and formation in vitro. **A**, **B** KYSE30, A2780, and HGC-27 cells were treated with gilteritinib at different concentrations for 24 h, and then stained using the EdU assay. **C**, **D** KYSE30, A2780, and HGC-27 cells were treated with the indicated concentrations of gilteritinib for 7 days, and the resulting cell colonies were fixed and stained with crystal violet and counted. **E**–**G** Cell spheroids (KYSE30-s, A2780-s, and HGC-27-s) were treated with different concentration of gilteritinib for 72 h. **E** Morphological alterations in KYSE30-s, A2780-s, and HGC-27-s cells 72 h after gilteritinib treatment using phase contrast microscopy. **F** The cell viability of spheroids was monitored using the Cell Titer-Glo® Luminescent Cell Viability Assay. **G** Fluorescence microscopy images showing the viability of KYSE30-s, A2780-s, and HGC-27-s cells cultured in vitro with FDA (Fluorescein diacetate) stain. Scale bars: 100 µm. Data are presented as the mean ± standard error of the mean (SEM); the statistical significance was determined by unpaired *t*-test (*p < 0.05; **p < 0.01).

To further explore the antitumor potential of gilteritinib and the underlying mechanisms, we treated KYSE30, A2780, and HGC-27, representing EC, OC, and GC, respectively, with gilteritinib. The results of the EdU staining assay (Fig. 2A, B) and clonogenic assay (Fig. 2C, D) indicated that gilteritinib dosage-dependently inhibited cell proliferation in KYSE30, A2780, and HGC-27 cells. Gilteritinib exhibited excellent antiproliferative activity against AXL-positive solid tumors in vitro.

Cancer stem cells (CSCs) represent a subpopulation of cells that display stem cell characteristics, which have been recently implicated in self-renewal, drug resistance, and metastatic potential, thus adversely affecting the outcomes of patients with cancer [25–27]. Cancer cell spheroid formation in suspension culture is a prominent characteristic of CSCs and can be used for in vitro evaluation [28–30]. To investigate the impact of gilteritinib treatment on the spheroid-forming capacity, morphology, and viability of KYSE30, A2780, and HGC-27 cells were cultured to form spheroids (KYSE30-s, A2780-s, and HGC-27-s, respectively). Our data indicated that the number and size of spheroids significantly and dose-dependently decreased with increasing concentrations of gilteritinib compared to the untreated spheroids (Fig. 2E). Doses-dependent death of cancer cell spheroids was confirmed by the Cell TiterGlo Luminescent Cell Viability assay (Fig. 2F). In addition, FDA (Fluorescein diacetate) staining demonstrated that gilteritinib markedly and dose-dependently increased cell death relative to the untreated group in KYSE30-s, A2780-s, and HGC-27-s cells (Fig. 2G). Together, these results indicate that gilteritinib significantly inhibits cell proliferation and spheroid formation in vitro.

### Gilteritinib markedly induces EC, OC, and GC cell cycle arrest

**Fig. 3:**
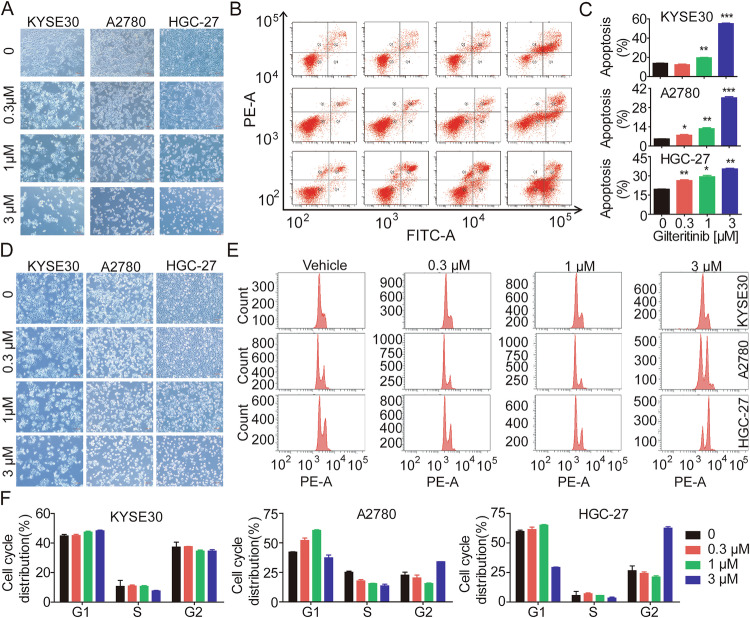
Gilteritinib induces apoptosis and cell-cycle arrest in vitro. **A**–**C** KYSE30, A2780, and HGC-27 cells were treated with 0, 0.3, 1, and 3 μM gilteritinib for 24 h, before analyzing the proportion of apoptotic cells by flow cytometry, with the percentage of Annexin V/PI-positive cells depicted in bar charts. **D**–**F** Cells were treated with 0, 0.3, 1, and 3 μM gilteritinib for 24 h, before determining the cell cycle by flow cytometry, with the cell cycle distributions depicted in bar charts. Scale bars: 100 µm. Data are presented as the mean ± standard error of the mean (SEM); the statistical significance was determined by unpaired t-test (*p < 0.05; **p < 0.01; ***p < 0.001).

To determine the mechanism by which gilteritinib exerted its antiproliferative activity against AXL-expressing solid tumor cells, we investigated the cell cycle profile and apoptosis of KYSE30, A2780, and HGC-27 cells. After treatment with gilteritinib for 24 h, the cell survival was noticeably reduced in a dose-dependent manner compared to that in the vehicle group (Fig. 3A,D). We next evaluated the effect of gilteritinib on apoptosis, with Annexin V-AAD staining, which revealed that gilteritinib significantly and dose-dependently induced apoptosis in KYSE30, A2780, and HGC-27 cells (Fig. 3A–C). Treatment of KYSE30 and A2780 cells with gilteritinib induced significant cell cycle arrest, characterized by a decrease in G0–G1 phase cells and an increase in G2-M-phase cells (Fig. 3D–F). However, no significant cell cycle arrest was observed in KYSE30 cells. Gilteritinib exerted significant antitumor effects by inducing apoptosis and cell cycle arrest in AXL-expressing EC, OC, and GC cells.

### Gilteritinib predominantly suppresses EC, OC, and GC cell motility

**Fig. 4:**
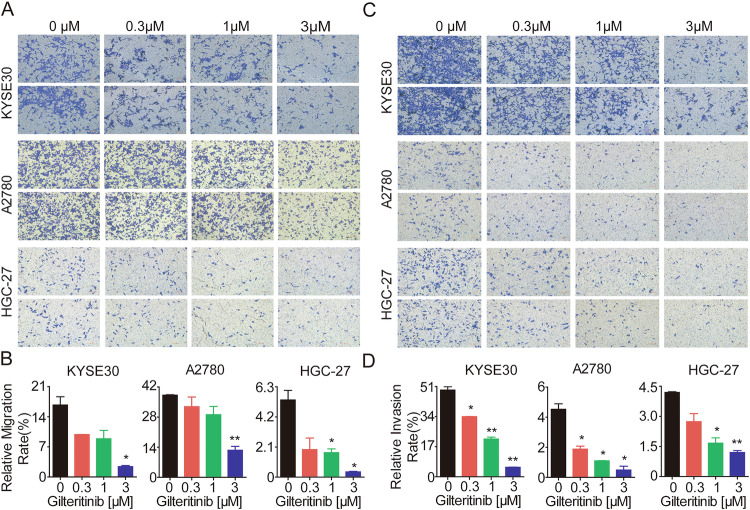
Gilteritinib suppresses cell motility in vitro. KYSE30, A2780, and HGC-27 cells were exposed to 0, 0.3, 1, and 3 μM gilteritinib for 24 h. The cell motility was detected by Transwell migration (**A**, **B**) and invasion assays (**C**, **D**). Data are presented as the mean ± standard error of the mean (SEM); the statistical significance was determined by unpaired *t*-test (*p < 0.05; **p < 0.01). Scale bars: 100 µm.

Tumor cell dissemination is one of the basic characteristics of malignant tumors and is the main cause of tumor recurrence and distant metastasis, thus seriously influencing the prognosis and survival of patients with cancer [31, 32]. Next, we examined the effect of gilteritinib on the motility of AXL-expressing EC, OC, and GC cells. KYSE30, A2780, and HGC-27 cells were treated with gilteritinib and detected using Transwell assays. Compared to the untreated group, gilteritinib dose-dependently inhibited the migration (Fig. 4A, B) and invasion (Fig. 4C, D) abilities of KYSE30, A2780, and HGC-27 cells, revealing that gilteritinib significantly suppressed the metastasis capacity of AXL-positive EC, OC, and GC cells.

### Effects of gilteritinib on global gene expression profiles of EC, OC, and GC cells

**Fig. 5:**
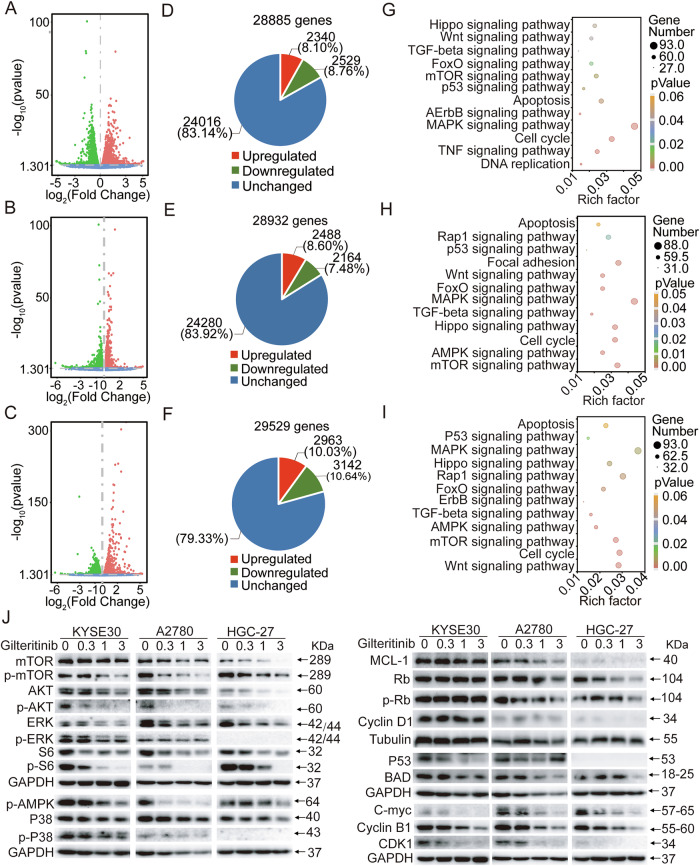
Effects of gilteritinib treatment on the global gene expression profile. Gene expression studies were conducted by RNA-seq in KYSE30, A2780, and HGC-27 cells treated with vehicle control or 1 μM gilteritinib for 24 h. Each group represents triplicate samples. The expression of DEGs in KYSE30 (**A**), A2780 (**B**), and HGC-27 (**C**) cells between the vehicle and gilteritinib treatment. Upregulated and downregulated genes are shown in red and green, respectively. Values are presented as the log10 of tag counts. Number of overlapping DEGs in KYSE30 (**D**), A2780 (**E**), and HGC-27 (**F**) treated cells as compared to the vehicle-treated cells. Kyoto Encyclopedia of Genes and Genomes (KEGG) analysis of DEGs in the gilteritinib treated samples as compared to the vehicle-treated samples in KYSE30 (**G**), A2780 (**H**), and HGC-27 (**I**) cells. **J** Analysis of the PI3K/AKT pathway, AMPK pathway, p53 pathway, cell cycle-related proteins, and antiapoptotic proteins, with GAPDH or β-tubulin used as the loading control.

To further unravel the potential mechanisms underlying gilteritinib treatment, bulk RNA-seq was conducted in KYSE30, A2780, and HGC-27 cells treated with gilteritinib. We analyzed the differentially expressed genes (DEGs) in each group using multidimensional scaling (Fig. 5A–C). In these cells, a significant number of genes exhibited differential expression in the gilteritinib-treated group compared to the untreated group. Specifically, in the treated group, 4652 (16.08%), 6105 (20.67%), and 4869 (16.86%) genes were significantly differentially expressed in KYSE30, A2780, and HGC-27 cells, respectively. Among these genes, transcripts of 2488 (8.60%), 2963 (10.03%), and 2340 (8.10%) genes were upregulated, whereas transcripts of 2164 (7.48%), 2963 (10.64%), and 2529 (8.76%) genes were downregulated compared to the control group, respectively.

Kyoto Encyclopedia of Genes and Genomes (KEGG) analysis of the DEGs demonstrated that multiple pathways were regulated by gilteritinib treatment in KYSE30, A2780, and HGC-27 cells. KEGG analysis of the 4652 (16.08%), 6105 (20.67%), and 4869 (16.86%) genes significantly modulated by gilteritinib indicated that gilteritinib primarily modulated signaling pathways associated with apoptosis, cell cycle, the mTOR pathway, AMPK pathway, p53 pathway, FOXO pathway, Hippo pathway, and Wnt pathway, which were essential for cancer cell proliferation and metastasis. Additionally, the prominent genes associated with these pathways were significantly altered in the gilteritinib-treated KYSE30, A2780, and HGC-27 cells (Fig. 5D–F and Supplemental Figs. 1–3).

We further validated the effects of gilteritinib on the biological processes and related signaling pathways mentioned above in KYSE30, A2780, and HGC-27 cells. Consistent with the RNA-Seq analysis, gilteritinib decreased the phosphorylation of mTOR, AKT, ERK, S6, P38, and AMPK, indicating that gilteritinib inhibited these pathways in all three cell lines. In addition, the protein levels of BAD, c-Myc, p53, Cyclin B1, Cyclin D1, and CDK1 were reduced by gilteritinib. The changes in protein expression related to the cell cycle and apoptosis also resulted in cell cycle arrest and inhibition of KYSE30, A2780, and HGC-27 cell proliferation (Fig. 5J).

Collectively, these data clearly demonstrate that gilteritinib inhibits multiple signaling pathways, including the cell cycle and apoptosis, to exert its antitumor effects in AXL-positive EC, OC, and GC cells in vitro.

### Gilteritinib treatment affects additional pathways and downregulated unique prognostic genes associated with EC, OC, and GC

**Fig. 6:**
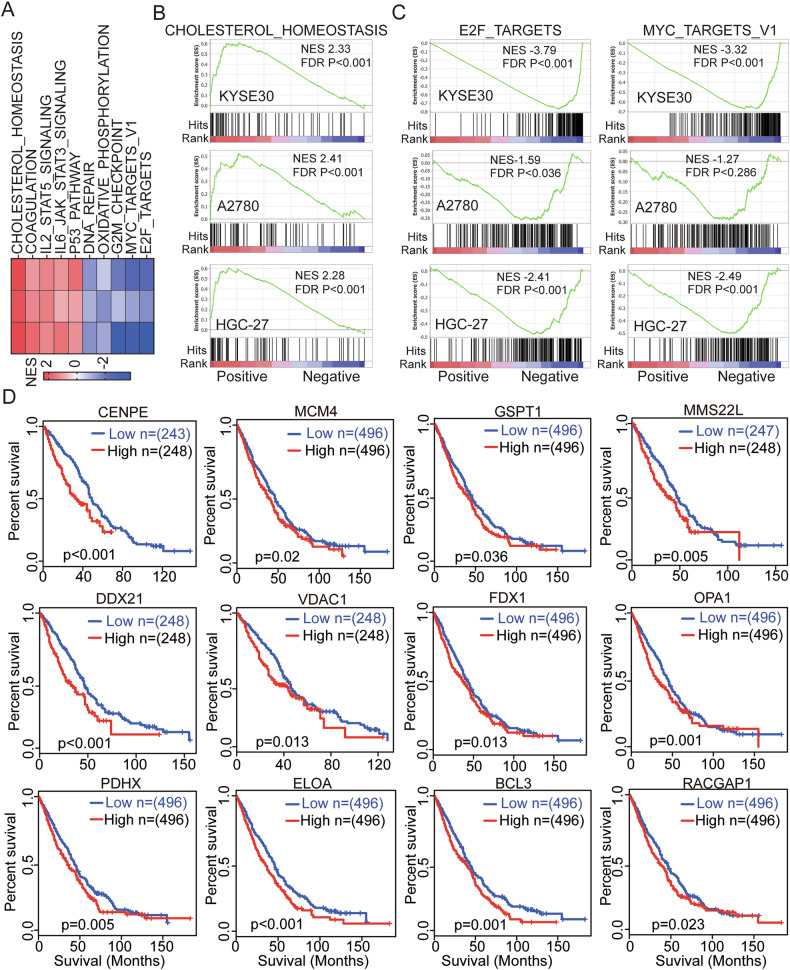
Gilteritinib treatment affects additional pathways and downregulates unique EC-, OC-, and GC-associated prognostic genes. **A** The top ranked positively and negatively enriched gene sets identified using Gene Set Enrichment Analysis (GSEA) in response to gilteritinib treatment. GSEA was conducted in KYSE30, A2780, and HGC-27 cells after gilteritinib treatment using the 50 HALLMARK gene sets database in MSigDB. GSEA plots showing strong positive enrichment of the cholesterol_homeostasis pathway (**B**), negative enrichment of the E2F and MYC targets v1 (**C**) in KYSE30, A2780, and HGC-27 cells in response to gilteritinib. NES Normalized enrichment score, FDR False discovery rate. **D** Kaplan–Meier estimate of overall survival based on DEG expression (TCGA EC, OC, and GC cohort). Relative high expression of *CENPE*, *MCM4*, *GSPT1*, *MMS22L*, *DDX21*, *VDAC1*, *FDX1*, *OPA1*, *PDHX*, *ELOA*, *BCL3*, and *RACGP1* genes was associated with poor overall survival in the EC, OC, and GC cohort. Log rank (Mantel-Cox) test was used for significance; *p* < 0.05 was considered significant.

Next, Gene Set Enrichment Analysis (GSEA) was conducted using the 50 Hallmark gene set collections in the MSigDB for the identification of specifically enriched biological pathways following gilteritinib treatment. GSEA of the common DEGs in KYSE30, A2780, and HGC-27 cells treated with gilteritinib revealed strong positive enrichment of gene sets involved in hallmark_cholesterol_homeostasis (Fig. 6A, B). Strong negative enrichment for the gene sets involved in cell cycle progression and proliferation, including hallmark_E2F_targets and hallmark_MYC_ targets_V1, was also observed (Fig. 6A–C). The upregulated and downregulated gene sets are presented in Fig. 6A and Supplemental Table 2.

To validate the biologic relevance of the genes in EC, OC, and GC regulated by gilteritinib, we explored TCGA dataset from the EC, OC, and GC cohorts to confirm whether any of the top 200 (100 up and down) DEGs identified by RNA-seq analysis were aberrantly expressed and/or associated with outcome. As a result, 12 genes were found to be downregulated following gilteritinib therapy, including CENPE, MCM4, GSPT1, MMS22L, DDX21, VDAC1, FDX1, OPA1, PDHX, ELOA, BCL3, and RACGP1, in EC, OC and GC TCGA cohort (Supplemental Fig. 4). Kaplan–Meier survival analysis indicated that high expression of each of these genes was associated with poor overall survival in the EC, OC, and GC TCGA cohorts (*p* < 0.05) (Fig. 6D). Intriguingly, the genes that were downregulated in response to gilteritinib, also appear to be overexpressed and associated with poor prognosis in the EC, OC, and GC TCGA cohorts (CENPE, MCM4, GSPT1, MMS22L, DDX21, and VDAC1) that were components of the most enriched pathways, such as E2F and MYC, identified by GSEA.

### Gilteritinib effectively induces cell death in EC, OC, and GC cells in the histoculture drug sensitivity test (HDST)

**Fig. 7:**
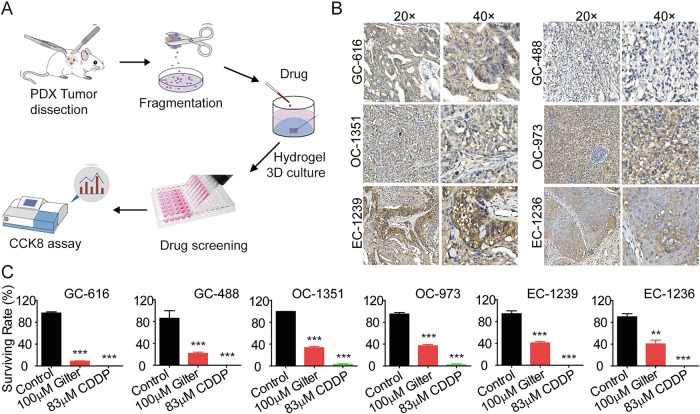
Gilteritinib effectively inhibits tumor growth in the HDST model ex vivo. **A** HDST analysis was used to assess the therapeutic effect of gilteritinib on different PDX derived explants (PDXE). The PDXE fragments submerged with hydrogel to form an ex vivo culture system. **B** The expression of AXL was analyzed by immunohistochemistry in the PDEXs from human GC samples (GC-616 and GC488), OCs (OC-1351 and OC-973), and EC samples (EC-1239 and EC-1236). The images were captured at 200× magnification (left) and 400× magnification (right). Scale bars: 50 µm (left) and 20 µm (right). **C** Cells from the indicated PDXE were treated with 100 mM gilteritinib and 83 mM CDDP (Cisplatin) as a positive control for 5 days, before detecting the cell viability by CCK-8. Data are presented as the mean ± standard error of the mean (SEM); the statistical significance was determined by unpaired *t*-test (**p < 0.01; ***p < 0.001).

It has been reported that the use of ex vivo patient-derived explants (PDEs) or PDX-derived explants (PDXEs) has many advantages over other preclinical model systems, such as immortalized cell lines. PDEs have been proven to predict patient response when exposed to therapeutic agents [33, 34]. To further evaluate the antitumor activity of gilteritinib on AXL-positive EC, OC, and GC cells, we next analyzed the clinically related human PDEXs from PDX mice by hydrogel-embedded HDST analysis (Fig. 7A). The IHC assay demonstrated that AXL was expressed in human GC (GC-616 and GC488), OC (OC-1351 and OC-973), and EC (EC-1239 and EC-1236) PDEXs (Fig. 7B). Six PDXE tissue samples were treated with 100 μM gilteritinib, with 83 μM CDDP serving as the positive control. Our results showed that gilteritinib significantly induced antitumor effects in AXL-positive EC, OC, and GC PDEX cells (Fig. 7C).

In summary, these exciting findings demonstrated that gilteritinib significantly and effectively improved tumoricidal activity ex vivo. This finding provides a strong rationale for the subsequent clinical development of gilteritinib for treating patients with AXL-positive EC, OC, and GC.

### Gilteritinib significantly inhibits advanced EC PDXO growth

**Fig. 8:**
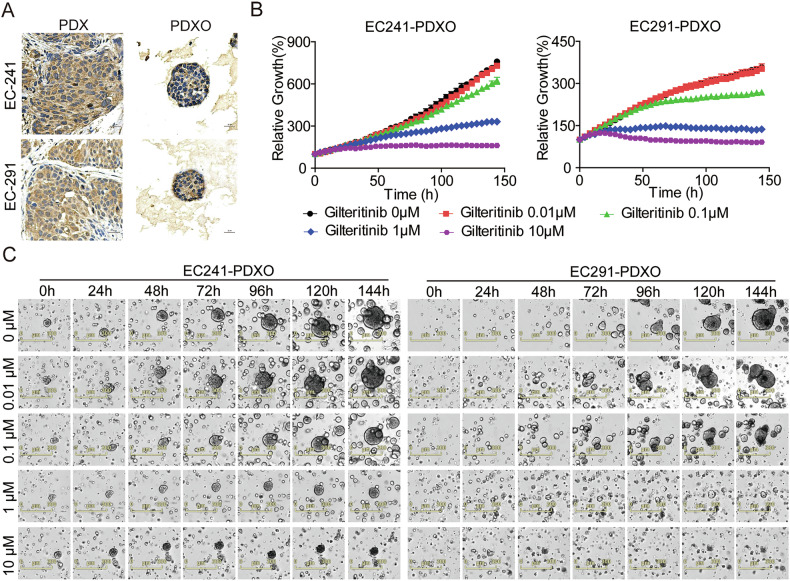
Inhibitory effects of gilteritinib on EC PDXO growth. **A** IHC staining of AXL expression in EC PDXs and their corresponding PDXOs. DAPI nuclear stain is shown in blue. Images were captured at 400× magnification. Scale bars: 20 µm. **B** EC241 and EC291 PDXO models were treated with the indicated concentrations of gilteritinib from 0 h to 144 h. The organoid size (%) was calculated using Incucyte S3 Zoom software based on phase-contrast images. **C** Brightfeld images of PDXOs treated with gilteritinib from 0 h to 144 h. Scale bar: 300 μm. Each data point represents triplicate wells.

Patient-derived xenograft (PDX), patient-derived organoid (PDO), or PDX tumor-derived organoid (PDXO) models maintain the principal pathologic and genetic characteristics of their original tumors and have emerged as an alternative method to model patient diseases, leading to their wide use in preclinical drug development [35–39]. We established EC PDXO models derived from corresponding PDX tumors. IHC assays demonstrated that AXL was overexpressed and exhibited similar subcellular localization in PDXOs and their corresponding PDX tissues (Fig. 8A). Subsequently, we further evaluated the inhibitory effects of gilteritinib in EC241 and EC291 PDXOs using the IncuCyte® S3 live-cell imaging system. Live-cell imaging analysis demonstrated that gilteritinib inhibited growth in a dose- and time-dependent manner compared to the vehicle from 0 to 144 h in the two PDXOs (Fig. 8B, C). These results indicate that gilteritinib treatment displays synergistic anti-tumor effects in PDXOs, providing more comprehensive therapeutic efficacy ex vivo, with the potential to guide the selection of PDX models in vivo.

### Gilteritinib significantly improves antitumor activity in advanced EC PDX models

**Fig. 9:**
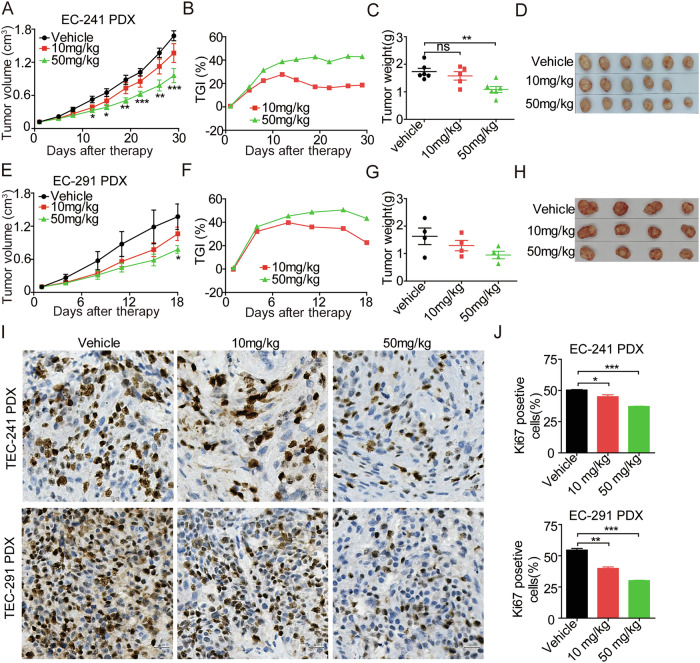
Antitumoral effects of gilteritinib in EC PDX models. Gilteritinib (10 mg/kg and 50 mg/kg) was administered orally once a day for 3–4 weeks. Antitumoral effects of gilteritinib on tumor growth in EC241 (**A**–**D**) and EC291 (**E**–**H**) PDX models. **I**, **J** IHC staining of Ki-67 in paraffin-embedded tumor tissue samples from the EC241 and EC291 PDX models. Images were captured at 400× magnification. Scale bars: 20 µm. Data represent the mean ± standard error of the mean (SEM) of at least three independent experiments; the statistical significance was assessed by unpaired *t*-test (ns Not significant; *p < 0.05; **p < 0.01; ***p < 0.001).

To further investigate the therapeutic efficacy of gilteritinib, two EC PDX models were evaluated. In the EC241 PDX model, gilteritinib showed dose-dependent antitumoral effects at doses of 10 and 50 mg/kg compared to the vehicle group. Gilteritinib at 50 mg/kg significantly improved the therapeutic effect compared to the vehicle group, resulting in a terminal tumor growth inhibition (TGI) of 42.9%. Treatment with 10 mg/kg gilteritinib led to a moderate decrease in tumor volume, resulting in TGIs of 18.62%, although this difference was not statistically significant (Fig. 9A–D). In the EC291 PDX model, gilteritinib showed antitumoral effects, as demonstrated by a reduction in tumor volume compared to the vehicle, and TGIs of 22.65% and 43.21% were observed on day 18 at doses of 10 mg/kg and 50 mg/kg, respectively. However, this difference was not statistically significant (Fig. 9E–H).

EC PDX xenograft tumors and important organs were then harvested for molecular and pathological analysis. Treatment with 10 and 50 mg/kg gilteritinib significantly reduced the number of Ki67-positive cells compared to the vehicle control (Fig. 9I, J). Gilteritinib treatment was well tolerated in the EC PDX models, with no significant changes in body weight observed (Supplementary Fig. 5). Additionally, there were no apparent toxic side effects on the heart, liver, spleen, lung, and kidney in any treatment group compared to the vehicle control, suggesting that this therapy had minimal in vivo toxicity in these PDX models (Supplementary Fig. 6).

In conclusion, our data demonstrate that treatment with gilteritinib improves therapeutic efficacy in vivo, providing a strong rationale for the subsequent clinical development of gilteritinib for treating patients with solid tumors.

## Discussion

AXL has recently emerged as a promising therapeutic target for the treatment of many cancer types. AXL inhibitors are currently in clinical development; thus, there is a strong need to systematically explore their antitumor effects and potential cellular mechanisms of action to avoid possible side effects and benefit more cancer patients. Gilteritinib is FDA-approved, orally available inhibitor that targets both FLT3 and AXL for treating patients with FLT3-mutated AML [18, 40]. However, the antitumor activity of gilteritinib in solid tumors remains poorly elucidated.

In this study, we first characterized AXL and FLT3 expression in human solid tumor cell lines and PDX tissues in response to gilteritinib. We found that EC, OC, and GC cells could express AXL using western blotting and immunofluorescence assays. All tested solid tumor cells expressed AXL, with high levels observed in TE-1, KYSE30, and A2780 cells, and relatively low levels in MKN45 cells. These findings confirm previous reports of AXL overexpression in EC, OC, and GC cells [7]. However, FLT3 expression was not detected in these cells. Consistent with reported data, FLT3 is almost exclusively expressed in the hematopoietic compartment, and the FLT3-ITD mutation level has been used as a prognostic marker for the treatment of patients with AML [41–43].

We determined the tumoricidal effects of the AXL inhibitor gilteritinib on cell growth, apoptotic cell death, cell cycle arrest, migration, and invasion in AXL-positive EC, OC, and GC cells in vitro and ex vivo. We present evidence that gilteritinib inhibits cell survival, triggers apoptosis and cell cycle arrest, and reduces cell motility, further suggesting that gilteritinib has therapeutic potential for tumor activity in AXL-positive solid tumor cells. Notably, the therapeutic potential of gilteritinib was further validated by ex vivo and in vivo studies, which demonstrated significantly improved antitumor effects in EC PDXO and PDX models treated with gilteritinib compared to the vehicle group.

To elucidate the mechanisms underlying the observed antitumor activity of gilteritinib in AXL-positive EC, OC, and GC cells, we assessed its effects on apoptosis and the cell cycle. Gilteritinib significantly and dose-dependently induced apoptosis and cell cycle arrest in KYSE30, A2780, and HGC-27 cells, as evidenced by the regulation of apoptotic protein expression (e.g., cleaved-PARP, BCL-2) and cell cycle regulatory protein expression (e.g., Rb, p-Rb, CyclinD1). Axl overexpression has been reported to promote tumor cell migration, invasion, and metastasis [44–46]. Our results revealed that gilteritinib significantly and dose-dependently repressed cell migration and invasion in AXL-positive EC, OC, and GC cells. In accordance with our data, a previous study demonstrated that silencing Axl in breast cancer cells could reduce lung metastasis in an orthotropic model [44, 46]. Taken together, these findings suggest that gilteritinib possesses strong antitumor activity, not only for treating primary solid tumors, but also for metastatic cancer.

Moreover, bulk RNA-seq data analysis further demonstrated that gilteritinib primarily affected genes related to apoptosis, the cell cycle, the mTOR pathway, the FOXO pathway, the Hippo pathway, and the Wnt pathway. Consistent with the RNA-seq analysis results, the phosphorylation levels of mTOR, AKT, ERK, S6, P38, and AMPK were downregulated by treatment with gilteritinib, as were the protein levels of BAD, P53, c-Myc, cyclin B1, and CDK1. These changes in protein expression related to the cell cycle and apoptosis supported the observed arrest and inhibition of cell proliferation. Additionally, global gene expression profiles and GSEA identified several important new gene sets that were regulated by gilteritinib therapy in KYSE30, A2780, and HGC-27 cells, with strong negative enrichment for the gene sets involved in cell cycle progression and proliferation, including hallmark_E2F_targets and hallmark_MYC_targets_V1. Intriguingly, a subset of six genes (CENPE, MCM4, GSPT1, MMS22L, DDX21, and VDAC1) is associated with poor prognosis in EC, OC, and GC when overexpressed. Of note, it has been reported that these genes are involved in tumor cell growth, proliferation, invasion, and metastasis in various cancers [47–54].

In conclusion, these exciting findings demonstrate that gilteritinib significantly and effectively induce tumoricidal activity in in vitro, ex vivo, and in vivo preclinical models. Gilteritinib induced strong antitumor activity by downregulating multiple cancer-related pathways, including those related to apoptosis, the cell cycle, mTOR pathway, AMPK pathway, P53 pathway, and FOXO pathway. These data provide a strong rationale for the subsequent clinical development of gilteritinib for the treatment of patients with AXL-positive solid tumors.

## Material and methods

### Drugs and reagents

Gilteritinib was purchased from Selleck (Houston, TX, USA).

### Cell lines

TE-1, KYSE30, HGC-27, and MKN45 were purchased from iCell Bioscience, Inc. (Shanghai, China). A2780 and SK-OV-3 were purchased from the American Type Culture Collection (Beijing, China) or OBiO Technology (Shanghai) Corp., Ltd. (Shanghai, China). TE-1, A2780, and MKN45 cells were cultured in RPMI-1640 medium (Gibco) supplemented with 10% fetal bovine serum (FBS) (Gibco). SK-OV-3 cells were cultured in McCoy’s 5 A medium (Gibco) supplemented with 10% FBS. HGC-27 cells were cultured in DMEM medium (Gibco) with 10% FBS. KYSE30 cells were cultured in a mixture of RPMI-1640 and F12 medium (Gibco) at a 1:1 ratio with 1% glutamine (Gibco) and 10% FBS and glutamine (Gibco). All of the abovementioned media were adjusted to include 1% penicillin/streptomycin (Biochem, Shenzhen, China). All cells were cultured in a humidified incubator (Thermo Fisher Scientific, Waltham, MA, USA) with 5% CO_2_ at 37 °C.

### In vitro cytotoxicity assay

EC, OC, and GC cells were seeded in 96-well plates at a density of 3000–5000 cells per well. Gilteritinib was added for 72 h, before determining the cell viability using a Cell Titer-Glo® luminescent cell viability assay kit (G7572, Promega, Madison, WI, USA) according to the manufacturer’s instructions. The luminescence was recorded using a SPARK Multiplate Reader (TECAN, Switzerland).

### IncuCyte S3 live-cell imaging system

EC, OC, and GC cells were treated with 10 μM gilteritinib for 72 h, before monitoring cell growth and confluence using the IncuCyte S3 live cell analysis system (Sartorius, Germany) and generating curves.

### Apoptosis and cell cycle arrest assays

KYSE30, A2780, and HGC-27 cells were seeded at a density of 2 × 10^5^ cells/well and exposed to different concentrations of gilteritinib for 24 h. Cell apoptosis was analyzed using an Annexin V-FITC Apoptosis Kit (Beyotime Biotechnology, Shanghai, China). The cell cycle was analyzed using a cell cycle assay kit (Beyotime Biotechnology).

### EdU staining assay

KYSE30, A2780, and HGC-27 cells were seeded on coverslips in 12-well plates at a density of 1 × 10^5^ cells/well before treatment with gilteritinib at various concentrations (0, 0.3, 1, and 3 μM) for 24 h. After treatment, the cells were stained using a BeyoClick™ EdU Cell Proliferation Kit (C0075S, Beyotime, Shanghai, China) according to the manufacturer’s instructions. Images were captured using a laser scanning confocal microscope (Zeiss880, Jena, Germany).

### Clonogenic assay

KYSE30, A2780, and HGC-27 cells were seeded on a 6-well plate at a density of 5000 cells/well and treated with 0, 0.3, 1, or 3 μM gilteritinib for 7 days. Cell colonies were washed and fixed with 4% paraformaldehyde for 30 min before staining with 0.01% (w/v) crystal violet for 15 min at room temperature. The stained colonies were scanned, and the total colony area was quantitated using ImageJ software.

### Cell migration and invasion assays

For the migration assay, KYSE30, A2780, and HGX-27 cells (2 × 10^4 ^cells/well) in serum-free medium were seeded into the upper chamber, and 600 μL medium containing 10% FBS was added to the lower chamber. Cells were incubated with gilteritinib at concentrations of 0, 0.3, 1, and 3 μM for 24 h. The migrated cells were fixed in 4% paraformaldehyde for 30 min and stained with crystal violet for 10 min at room temperature. The Transwell chambers were washed with distilled water and photographed under a light microscope. The migrated cells were counted using ImageJ software. The invasion assay was performed according to the method used for the migration assay, with the exception that the upper chamber was pre-coated with Matrigel before adding the cells.

### RNA-Seq analysis

Total RNA was isolated from KYSE30, A2780, and HGC-27 cells treated with gilteritinib for 24 h. The RNA integrity was assessed using the RNA Nano 6000 Assay Kit with the Bioanalyzer 2100 system (Agilent Technologies, CA, USA). Additionally, mRNA was purified from total RNA using poly-Toligo-attached magnetic beads, and the library fragments were purified using the AMPure XP system to select cDNA fragments of preferentially 370–420 bp in length. Next, the PCR products were purified using the AMPure XP system, and the library quality was assessed using the Agilent Bioanalyzer 2100 system. The clustering of the index-coded samples was performed on a cBot Cluster Generation System using a TruSeq PE Cluster Kit v3-cBot-HS (Illumia), according to the manufacturer’s instructions. After cluster generation, the library preparations were sequenced on an Illumina Novaseq platform, and 150-bp paired-end reads were generated.

### Western blot

KYSE30, A2780, and HGC-27 cells were incubated with gilteritinib at various concentrations (0, 0.3, 1, and 3 μM) for 24 h. Then, the cells were lysed using SDS lysis buffer (Beyotime Biotechnology, Shanghai, China) supplemented with PMSF (Solarbio, Beijing, China) and phosphatase inhibitor cocktail (Beyotime Biotechnology, Shanghai, China). Proteins were separated via SDS-PAGE and transferred to PVDF membranes (Millipore, Darmstadt, Germany). Next, the membranes were cut horizontally and incubated with primary and secondary antibodies. Protein bands were detected using ECL reagent (Cytiva, USA). Images were captured using a Bio-Rad Multifunctional chemiluminescence imaging system. The expression levels were normalized to GAPDH or β-tubulin and then compared to the control group. The antibody information is presented in Supplementary Table 1.

### Immunofluorescence

KYSE30, A2780, and HGC-27 cells were seeded on coverslips in 12-well plates at a density of 1 × 10^5^ cells/well. The cells were treated with gilteritinib for 24 h after adhesion. Next, the cells were fixed with 4% paraformaldehyde for 30 min. The cells were permeabilized with 0.1% Triton X-100 for 10 min, blocked with 5% bovine serum albumin for 1 h, and incubated with AXL primary antibodies at 4 °C overnight. The samples were then incubated with fluorescent secondary antibodies for 1 h at room temperature, and images were captured using a laser scanning confocal microscope (Zeiss880, Jena, Germany).

### Cell spheroids culture and viability assays

KYSE30, A2780, and HGC-27 cells were inoculated in 96-well round-bottom Ultra-Low Attachment surface spheroid microplates (Corning, USA) at a density of 2000 cells/well. The spheres formed from KYSE30, A2780, and HGC-27 cells were treated with gilteritinib for 72 h. Subsequently, the cell spheres were washed with phosphate buffered saline (PBS) three times after a 10-min incubation with fluorescein diacetate (FDA; Merck, Germany) operating solution at room temperature. Images were captured using a Leica fluorescence microscope (D-35578, Wetzlar, Germany). The viability of the cell spheres was determined using a Cell Titer-Glo® luminescent cell viability assay kit (G7572, Promega, Madison, USA), according to the manufacturer’s instructions. The luminescence was recorded using a SPARK Multiplate Reader (TECAN, Switzerland).

### Hydrogel-embedded histoculture drug sensitivity test

Clinical samples of EC, OC, and GC were used to establish patient-derived xenograft (PDX) mouse models (Nanchang Royo Biotech Co. Ltd). Mice with a tumor-bearing volume of 1000–1500 mm^3^ were selected, and their tumors were removed after euthanasia. Tumor tissue activity was detected using tissue activity dye, and inactive tissue was removed. Next, the tissue was soaked in tissue disinfectant for 1 min to disinfect it, followed by repeated cleaning with the tissue cleaning solution until the color was clear. Subsequently, the tumor tissue was divided into 2 × 2 × 2 mm^3^ tissue blocks and transferred to another clean Petri dish. The tissue was then pre-cooled on a metal ice bath and added to a 96-well deep-hole plate. The mixed hydrogel was added to each hole to form a glue and placed at room temperature for approximately 30 min. Subsequently, gilteritinib was added to groups according to the experimental purpose, with the inclusion of blank and positive control groups concurrently. After 5 days of cultivation, the drug-containing medium was sucked and cleaned with PBS three times. Glue + 10% CCK-8 of the total volume of the medium was added to the complete medium, and 150 μL of CCK-8 solution was added to each well after mixing. After incubation for 16–20 h, the solution was transferred to a new 96-well plate and the absorbance was measured at a wavelength of 450 nm.

### Organoid generation and viability assays

The PDX tumor tissue was cut into 0.5–2 mm^3^ small pieces and then washed three times in DPBS. Subsequently, the tissue was transferred to a 15-mL centrifuge tube, supplemented with 10 mL Tumor Tissue Digestion Solution (bioGenous, Hangzhou, China), and digested in an incubator at 37 °C for 1 h. After digestion was terminated using FBS, the tissue suspension was filtered through a 100-μm cell filter and centrifuged at 300 × *g* for 3 min. The supernatant was then removed, and the sediment was cleaned twice with Cancer Organoid Basal Medium (bioGenous, Hangzhou, China). The pellet was resuspended in TM Organoid Culture ECM (bioGenous, Hangzhou, China). Next, 50 µL of suspension in Matrigel was added to each well of a 24-well plate and incubated for 10 min at 37 °C for polymerization, before adding 500 µL of complete medium to each well. The organoids were then cultured in a humidified incubator with 5% CO_2_ at 37 °C. Next, to detect the viability of the organoids, the organoids were seeded in 96-well plates in a total volume of 150 µL of culture medium with 3 µL of Matrigel per well. After 24 h, EC organoids were treated with gilteritinib for 6 days. Finally, the organoid viability was detected using the IncuCyte S3 live cell analysis system (Sartorius, Gorteen, Germany).

### In vivo efficacy

For in vivo experiments, 5–6-week-old female NOD/SCID mice were provided by Jiangsu Huachuang Sino Pharma Tech Co., Ltd (Taizhou, China) and were housed and maintained under SPF (Specific Pathogen Free) conditions. All animal experiments were approved and performed in full accordance with the standards of the Biomedical Research Ethics Committee of Gannan Medical University (Jiangxi, China). Tumor tissues derived from EC PDX tissues were cut into to fragments of 2–3 mm^3^ and implanted subcutaneously into the flank of each mouse. When tumors reached approximately 100 mm^3^, the mice were randomized into vehicle control and treatment groups (n = 4–6). The mice in the vehicle control group were treated with DMSO, whereas the other two groups were treated with 10 or 50 mg/kg gilteritinib by gavage once daily. The tumor sizes and body weights were recorded twice weekly using electronic calipers, and tumor volumes were calculated using the following formula: tumor volume (mm^3^) = length × (width)^2^ × 0.5. T/C was defined as the treated tumor volume/control tumor volume × 100%, while the inhibition rate of tumor growth (TGI) was calculated as (1–T/C) × 100%. At the end of the experiments, the tumor volume did not exceed 1500 mm^3^, and the mice were sacrificed by euthanasia. The organs of the mice were collected for subsequent analyzes.

### Immunohistochemistry staining

Tumor tissue was fixed with 10% neutral formalin, dehydrated with a gradient alcohol, and embedded in paraffin. After cutting into sections of 4-µm thickness, the tissue slices were dewaxed using xylene and dehydrated using an alcohol gradient. Citric acid antigen retrieval solution was used for the AXL antibody, whereas the EDTA antigen retrieval solution was used for the Ki67 antibody. After antigen retrieval, the tissue sections were stained with an UltraSensitive^TM^ SP (mouse/rat) IHC Kit (MXBiotechnologies, Fuzhou, China), according to the manufacturer’s instructions. Stained sections were photographed using a panoramic tissue quantitative analysis system (Zeiss, Tissue FAXS PLUS).

### Hematoxylin and eosin (H&E) staining

The organs of mice were fixed with neutral formalin, dehydrated using an alcohol gradient, and embedded in paraffin. The organ slices, which had a thickness of 4 µm, were dewaxed using xylene and dehydrated using an alcohol gradient, after which they were stained with hematoxylin and eosin. The histological morphology and structure were observed using a panoramic tissue quantitative analysis system.

### Statistical analysis

All experiments were repeated at least three times. Data are expressed as the mean ± standard error of the mean (SEM) using GraphPad Prism 9 software. The IC50 values were calculated via nonlinear regression analysis of the concentration-response curves in SPSS 16.0. Statistical comparisons between two groups were performed using one-way ANOVA or Student’s *t*-test, with the untreated or vehicle control group as the reference. For all experiments, results with *P* < 0.05 were considered significant and are denoted in the figures as follows: *0.01 ≤ *P* < 0.05; **0.001 ≤ *P* < 0.01; and ***P < 0.001.

## Supplementary information

Western blot

Supplemental information

## Data Availability

The data used to support the findings of this study are available upon reasonable request from the corresponding author.
