# Supplementary figures and images for: Antitumor activity of gilteritinib, an inhibitor of AXL, in human solid tumors
Source: Cell Death Discov. 2025 Mar 29;11:124. doi: 10.1038/s41420-025-02417-9 (PMC11954984; doi:10.1038/s41420-025-02417-9)

Figure 1A

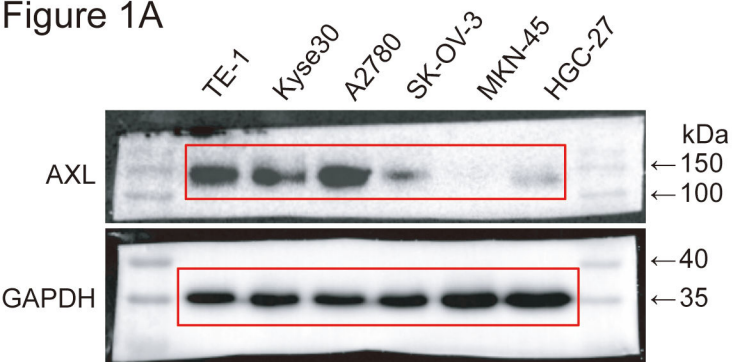

Figure 5J

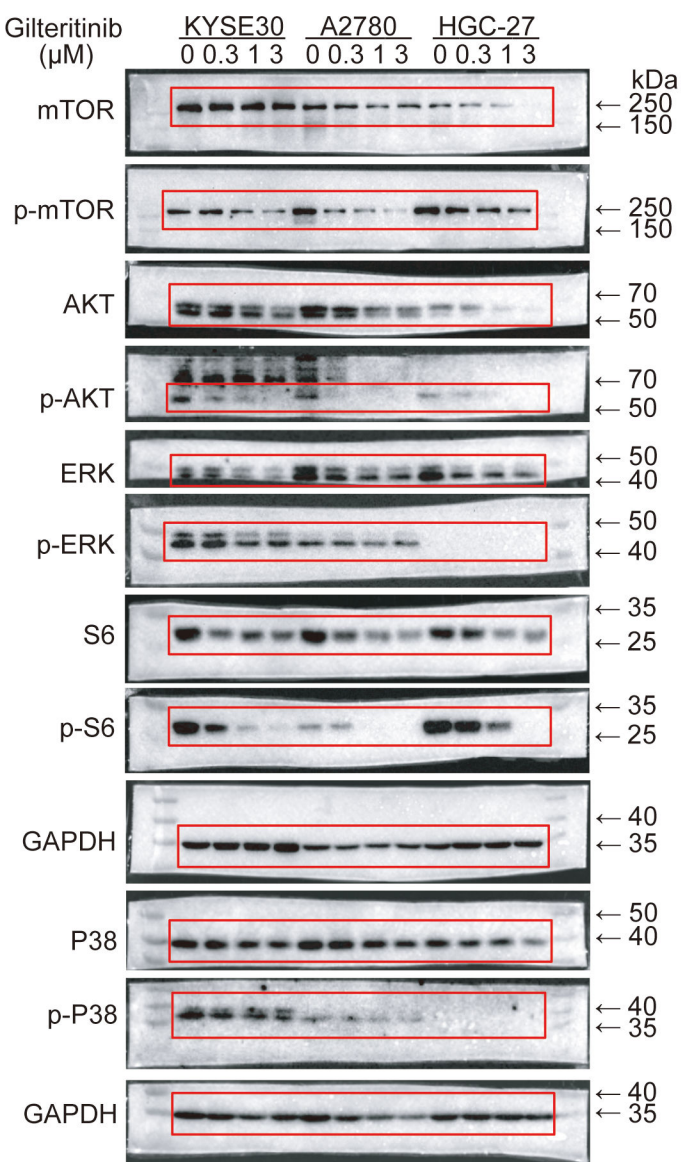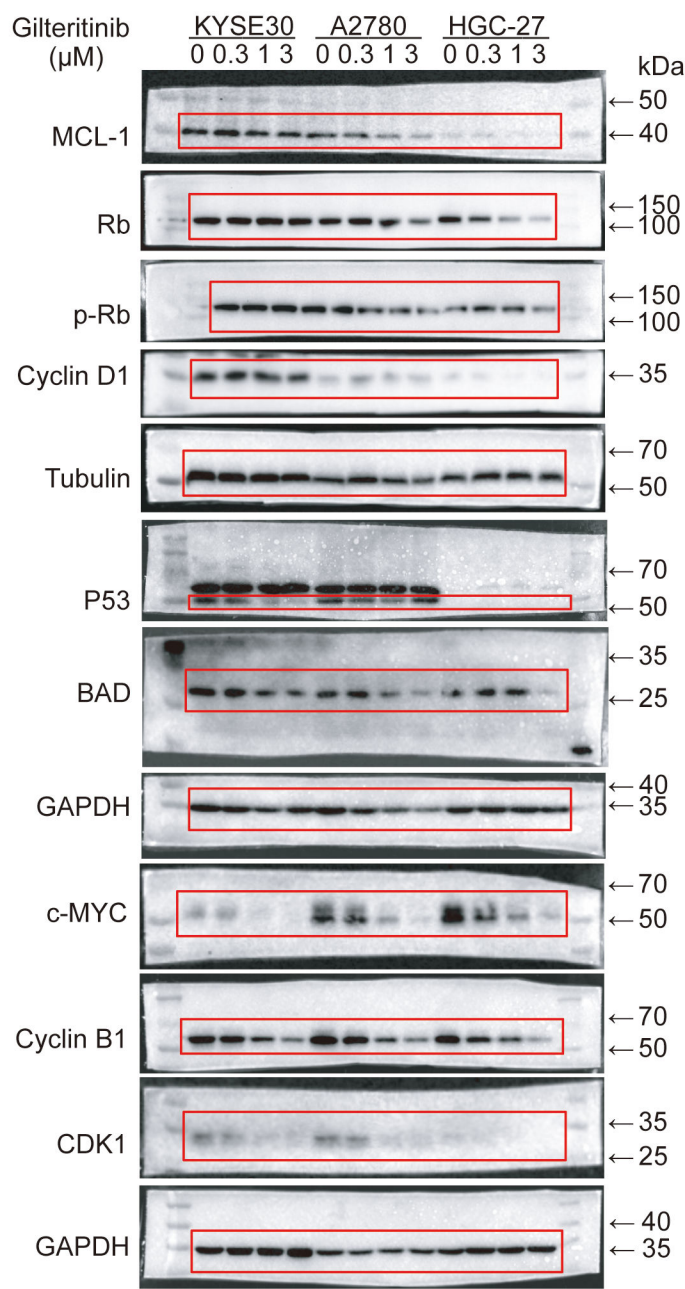

Supplement: Supplementary file 1 — Western blot [file 41420_2025_2417_MOESM1_ESM.pdf]
