# Supplementary material for: Antitumor activity of gilteritinib, an inhibitor of AXL, in human solid tumors
Source: Cell Death Discov. 2025 Mar 29;11:124. doi: 10.1038/s41420-025-02417-9 (PMC11954984; doi:10.1038/s41420-025-02417-9)
Supplement: Supplementary file 2 — Supplemental information [file 41420_2025_2417_MOESM2_ESM.docx]

# Supplemental information

# Supplemental Table 1. List of antibodies used for immunofluorescence, immunohistochemistry and western blot analysis.

| Target | Company | Cat No. | Application | Dilution |
| --- | --- | --- | --- | --- |
| AXL | proteintech | 13196-1-AP | IF/IHC | 1：500 |
| AXL | proteintech | 13196-1-AP | WB | 1：2000 |
| AKT | CST | 9272 | WB | 1：2000 |
| Phospho-Akt (Ser473) | CST | 4060 | WB | 1：2000 |
| mTOR | proteintech | 28273-1-AP | WB | 1：2000 |
| Phospho-mTOR (Ser2448) | CST | 2971 | WB | 1：1000 |
| p44/42 MAPK (Erk1/2) | CST | 4695 | WB | 1：2000 |
| Phospho-p44/42 MAPK (Erk1/2) (Thr202/Tyr204) | CST | 4370 | WB | 1：2000 |
| p38 MAPK | CST | 8690 | WB | 1：2000 |
| Phospho-p38 MAPK | CST | 4511 | WB | 1：2000 |
| S6 | CST | 2217 | WB | 1：2000 |
| Phospho-S6 | CST | 4858 | WB | 1：2000 |
| c-MYC | CST | 18583 | WB | 1：2000 |
| CDK1 | Affinity | AF6108 | WB | 1：2000 |
| Cyclin B1 | proteintech | 28603-1-AP | WB | 1：1000 |
| Cyclin D1 | proteintech | 26939-1-AP | WB | 1：1000 |
| Retinoblastoma | Affinity | AF6103 | WB | 1：2000 |
| Phospho-Retinoblastoma | Affinity | AF3103 | WB | 1：2000 |
| p53 | Proteintech | 10442-1-AP | WB | 1：2000 |
| BAD | Proteintech | 10435-1-AP | WB | 1：2000 |
| MCL-1 | CST | 94296 | WB | 1：2000 |
| GAPDH | CST | 5174 | WB | 1：10000 |
| Tubulin | Affinity | AF7011 | WB | 1：10000 |
| HRP-conjugated Affinipure Goat Anti-Rabbit IgG (H+L) | Proteintech | SA00001-2 | WB | 1：10000 |
| HRP-conjugated Affinipure Goat Anti-Mouse IgG (H+L) | Proteintech | SA00001-1 | WB | 1：10000 |

**Supplementary Table 2.** Gene set enrichment analysis (GSEA) analysis

| Gene set name | NES | NES | NES | FDR q VALUE | FDR q VALUE | FDR q VALUE |
| --- | --- | --- | --- | --- | --- | --- |
|  | KYSE30 | A2780 | HGC27 | KYSE30 | A2780 | HGC27 |
| [CHOLESTEROL_](http://www.gsea-msigdb.org/gsea/msigdb/human/geneset/HALLMARK_CHOLESTEROL_HOMEOSTASIS)  [HOMEOSTASIS](http://www.gsea-msigdb.org/gsea/msigdb/human/geneset/HALLMARK_CHOLESTEROL_HOMEOSTASIS) | 2.33 | 2.14 | 2.28 | 0.00 | 0.00 | 0.00 |
| COAGULATION | 1.84 | 1.32 | 1.09 | 0.00 | 0.12 | 0.37 |
| IL2_STAT5_  SIGNALING | 1.35 | 1.45 | 1.39 | 0.07 | 0.05 | 0.08 |
| IL6_JAK_STAT3_  SIGNALING | 1.66 | 0.84 | 1.14 | 0.01 | 0.98 | 0.30 |
| P53_PATHWAY | 1.17 | 0.96 | 1.71 | 0.24 | 0.71 | 0.01 |
| DNA_REPAIR | -1.56 | -1.01 | -1.51 | 0.02 | 0.75 | 0.02 |
| OXIDATIVE_  PHOSPHORYLATION | -1.14 | -1.59 | -0.64 | 0.28 | 0.05 | 0.99 |
| G2M_CHECKPOINT | -3.58 | -0.82 | -1.98 | 0.00 | 0.94 | 0.00 |
| MYC_TARGETS_V1 | -3.32 | -1.27 | -2.50 | 0.00 | 0.29 | 0.00 |
| E2F_TARGETS | -3.79 | -1.59 | -2.41 | 0.00 | 0.04 | 0.00 |

**
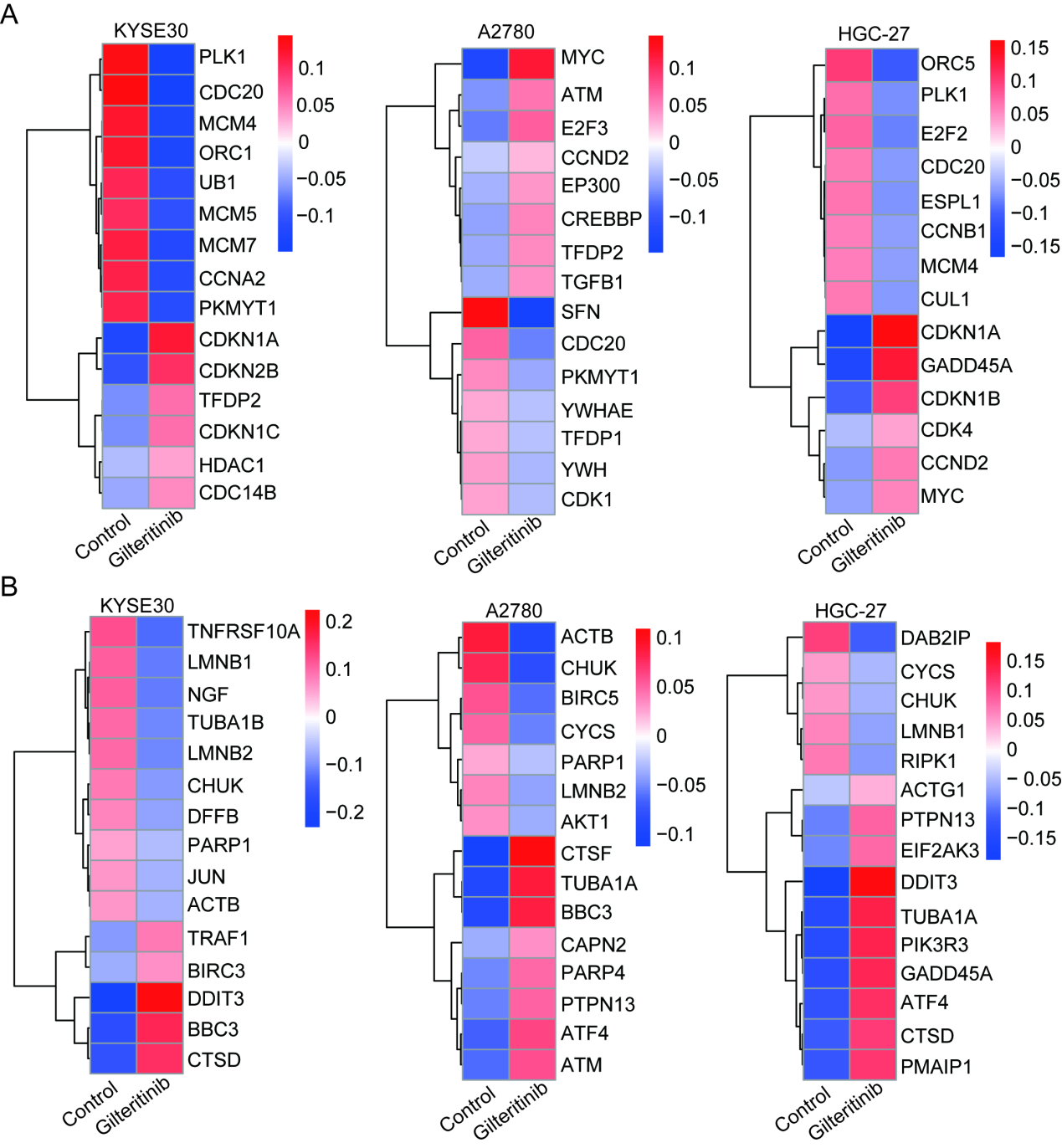
**

**Supplemental Figure 1.** Gilteritinib **significantly regulated the cell cycle and apoptosis DEGs expression in EC, OC and GC cells.** Heatmap significantly displaying the levels of major cell cycle (A) and apoptosis (B) related genes in the KYSE30, A2780 and HGC-27 cells treated with Control and 1 µM Gilteritinib.

**
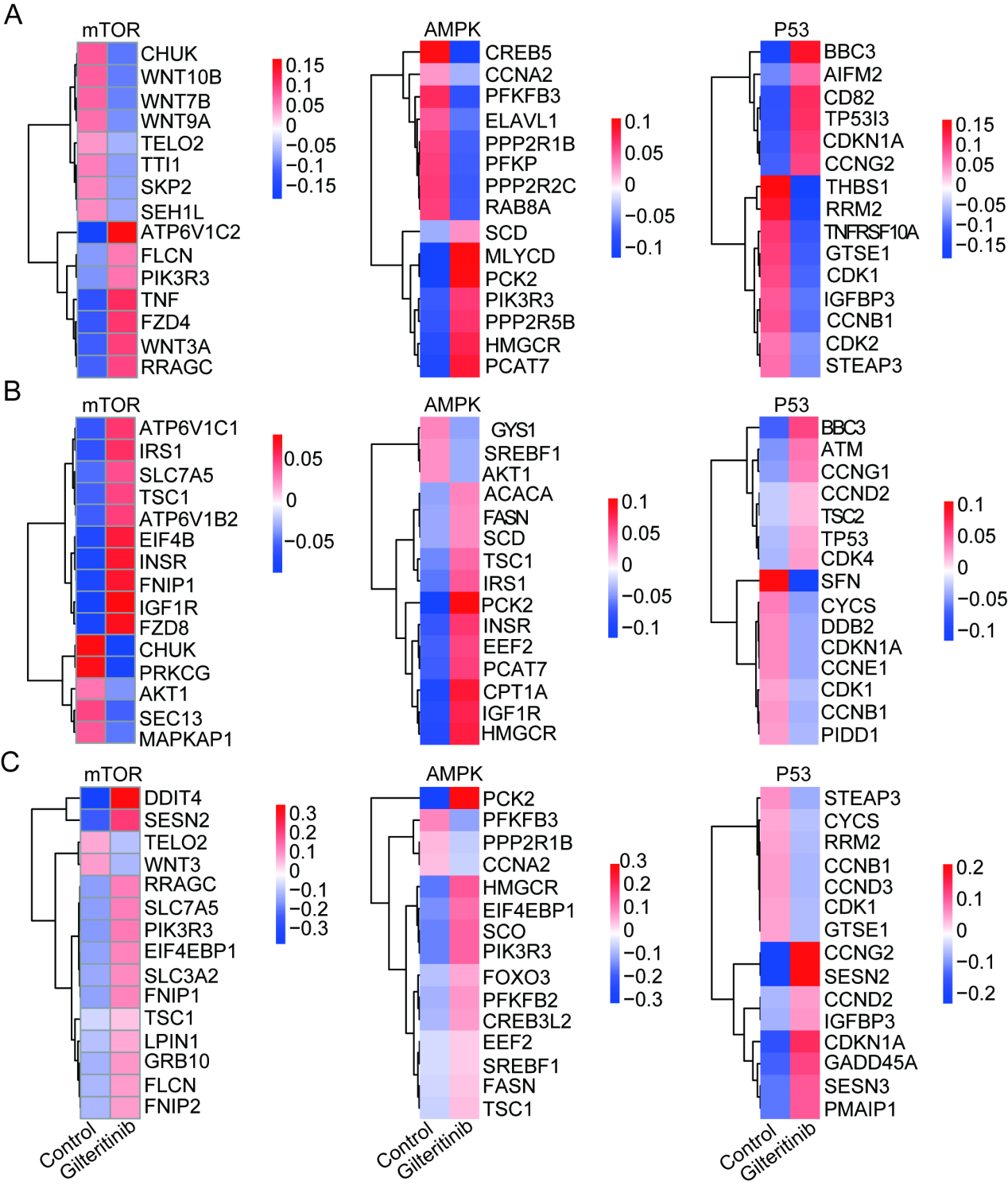
**

**Supplemental Figure 2.** Gilteritinib **significantly regulated the mTOR, AMPK and P53 pathway DEGs expression in EC, OC and GC cells.** Heatmap of significantly regulated genes of transcriptomes in the KYSE30 (A), A2780 (B) and HGC-27 (C) cells treated with Control and 1 µM Gilteritinib, correlated with the mTOR, AMPK and P53 signaling pathway.

**
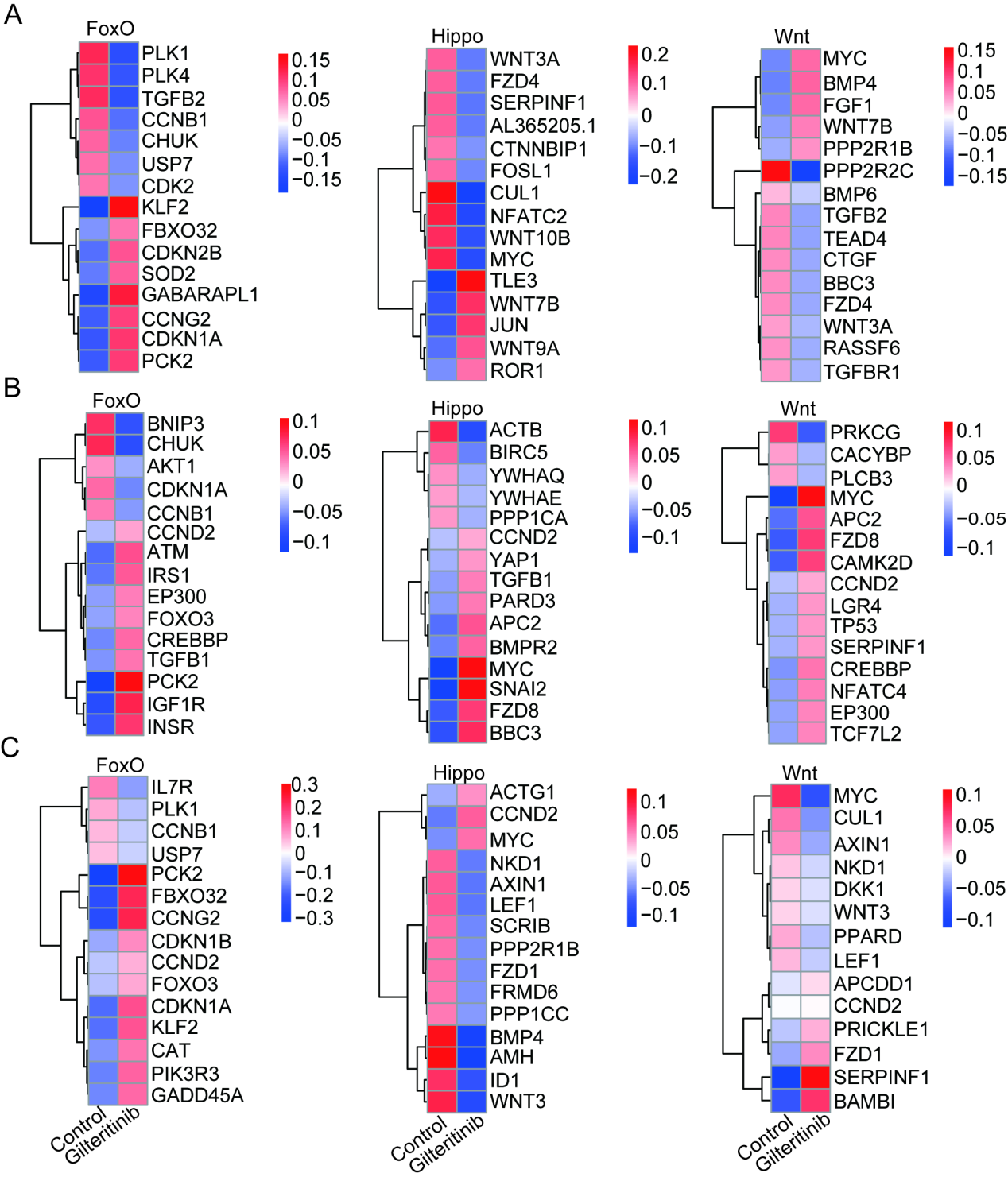
**

**Supplemental Figure 3.** Gilteritinib **significantly regulated the FOXO, Hippo and Wnt pathway DEGs expression in EC, OC and GC cells.** Heatmap of significantly regulated genes of transcriptomes in the KYSE30 (A), A2780 (B) and HGC-27 (C) cells treated with Control and 1 µM Gilteritinib, correlated with the FOXO, Hippo and Wnt signaling pathway.

**
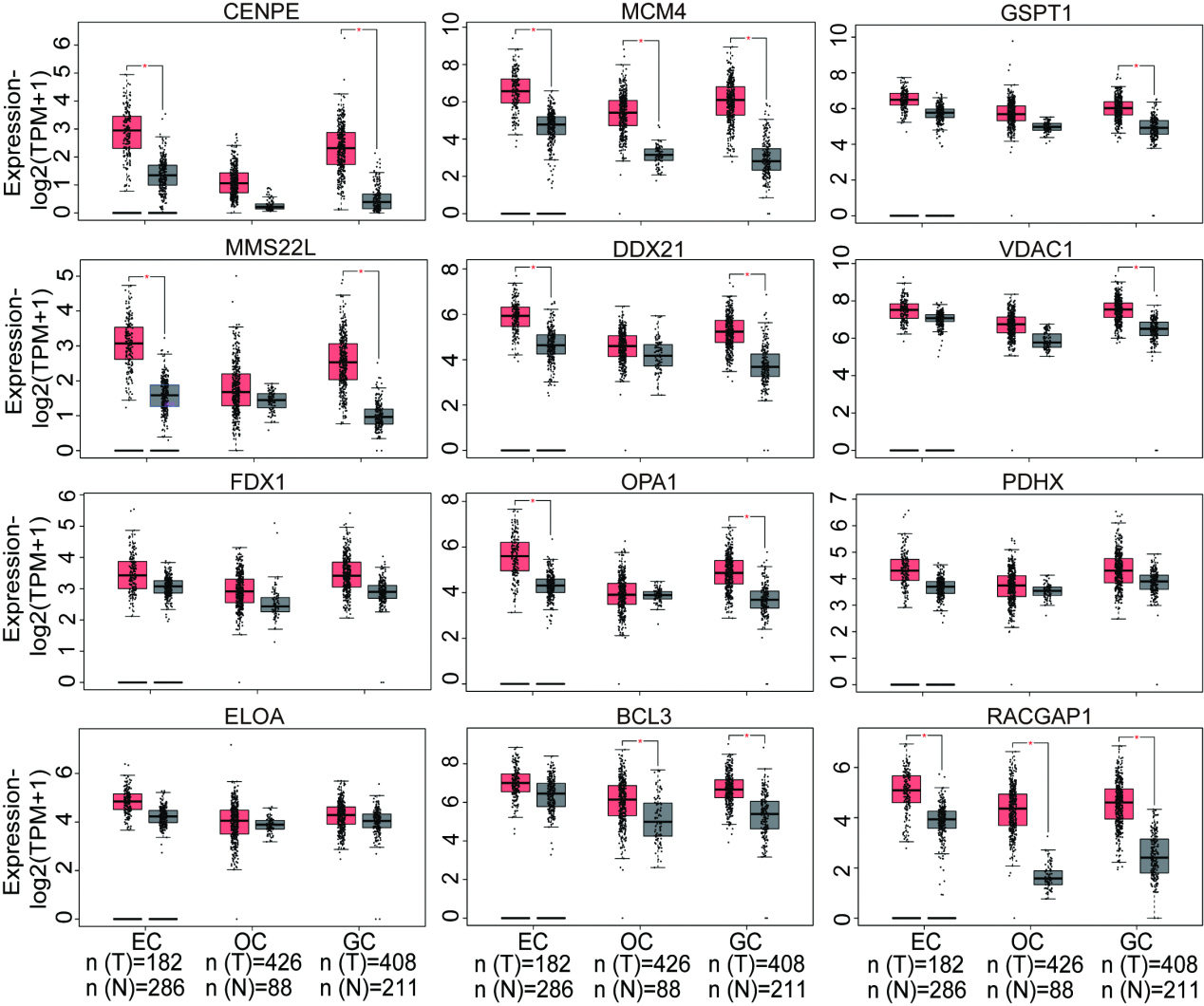
**

**Supplemental Figure 4. Expression of genes in EC, OC and GC TCGA cohort.** The expression of CENPE, MCM4, GSPT1, MMS22L, DDX21, VDAC1, FDX1, OPA1, PDHX, ELOA, BCL3 and RACGP1 genes were high in EC, OC and GC in comparison to normal controls. p<0.05 is considered significant and was calculated by the two tailed Student’s t test.


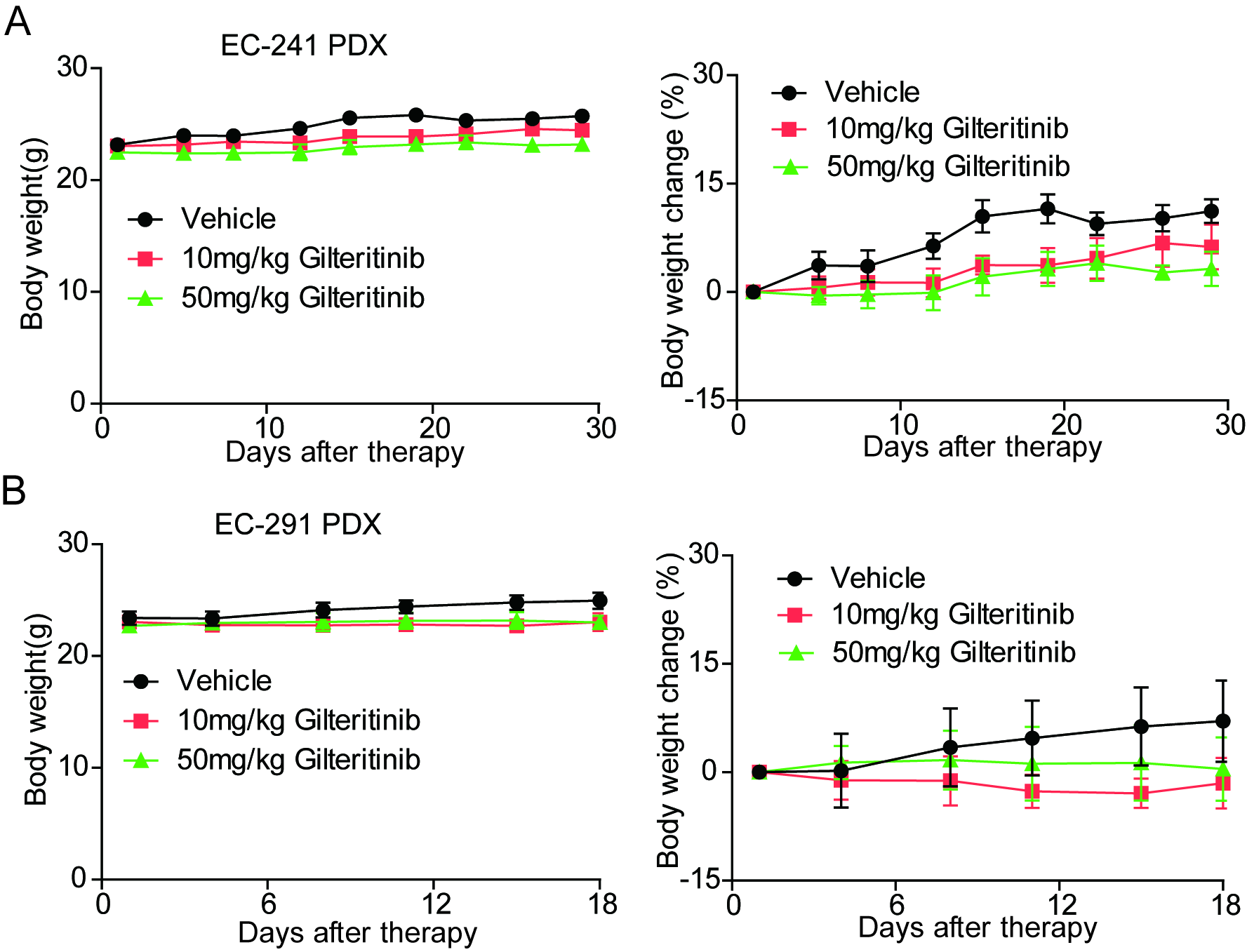


**Supplemental Figure 5. Effects of Gilteritinib on body weight of EC PDX models.** The weights and their corresponding weight changes of mice following treatment with Gilteritinib as indicated, were assessed twice every week in EC-241 (A) and EC-291 (B) PDX models.


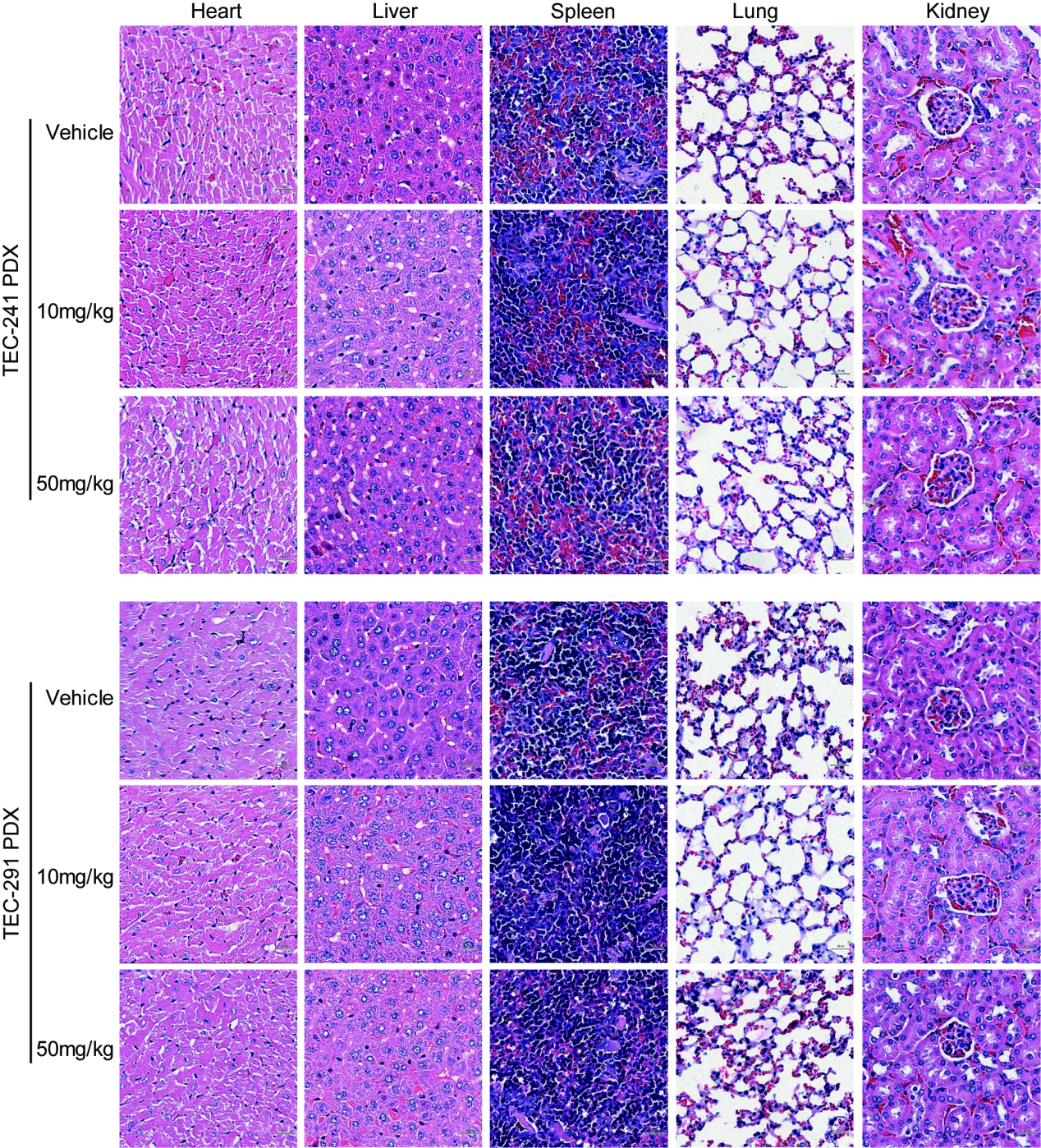


**Supplemental Figure 6. Effects of Gilteritinib on histology of EC PDX models.** Histopathology (H&E) of the vital organs (heart, liver, spleen, lung and kidney) of EC-241 and EC-291 PDXs at the end of the experiment. The images were scanned and captured using digital scanner EVOS Image system at 200 × magnification. Scale bars = 50 µm.
